# Hospital costs in relation to body-mass index in 1·1 million women in England: a prospective cohort study

**DOI:** 10.1016/S2468-2667(17)30062-2

**Published:** 2017-04-05

**Authors:** Seamus Kent, Jane Green, Gillian Reeves, Valerie Beral, Alastair Gray, Susan A Jebb, Benjamin J Cairns, Borislava Mihaylova, Million Women Study collaborators, Hayley Abbiss, Simon Abbott, Rupert Alison, Miranda Armstrong, Krys Baker, Angela Balkwill, Isobel Barnes, Valerie Beral, Judith Black, Roger Blanks, Kathryn Bradbury, Anna Brown, Benjamin Cairns, Dexter Canoy, Andrew Chadwick, Dave Ewart, Sarah Ewart, Lee Fletcher, Sarah Floud, Toral Gathani, Laura Gerrard, Adrian Goodill, Jane Green, Lynden Guiver, Alicia Heath, Darren Hogg, Michal Hozak, Isobel Lingard, Sau Wan Kan, Nicky Langston, Kath Moser, Kirstin Pirie, Alison Price, Gillian Reeves, Keith Shaw, Emma Sherman, Rachel Simpson, Helena Strange, Sian Sweetland, Sarah Tipper, Ruth Travis, Lyndsey Trickett, Anthony Webster, Clare Wotton, Lucy Wright, Owen Yang, Heather Young, Emily Banks, Valerie Beral, Lucy Carpenter, Carol Dezateux, Jane Green, Julietta Patnick, Richard Peto, Cathie Sudlow

**Affiliations:** a Cancer Epidemiology Unit, Nuffield Department of Population Health, University of Oxford, Oxford, UK; b Health Economics Research Centre, Nuffield Department of Population Health, University of Oxford, Oxford, UK; c Nuffield Department of Primary Care Health Sciences, University of Oxford, Oxford, UK

## Abstract

**Background:**

Excess weight is associated with poor health and increased health-care costs. However, a detailed understanding of the effects of excess weight on total hospital costs and costs for different health conditions is needed.

**Methods:**

Women in England aged 50–64 years were recruited into the prospective Million Women Study cohort in 1996–2001 through 60 NHS breast cancer screening centres. Participants were followed up and annual hospital costs and admission rates were estimated for April 1, 2006, to March 31, 2011, in relation to body-mass index (BMI) at recruitment, overall and for categories of health conditions defined by the International Classification of Diseases 10th revision chapter of the primary diagnosis at admission. Associations of BMI with hospital costs were projected to the 2013 population of women aged 55–79 years in England.

**Findings:**

1 093 866 women who provided information on height and weight, had a BMI of at least 18·5 kg/m^2^, and had no previous cancer at recruitment, were followed up for an average of 4·9 years from April 1, 2006 (12·3 years from recruitment), during which time 1·84 million hospital admissions were recorded. Annual hospital costs were lowest for women with a BMI of 20·0 kg/m^2^ to less than 22·5 kg/m^2^ (£567 per woman per year, 99% CI 556–577). Every 2 kg/m^2^ increase in BMI above 20 kg/m^2^ was associated with a 7·4% (7·1–7·6) increase in annual hospital costs. Excess weight was associated with increased costs for all diagnostic categories, except respiratory conditions and fractures. £662 million (14·6%) of the estimated £4·5 billion of total annual hospital costs among all women aged 55–79 years in England was attributed to excess weight (BMI ≥25 kg/m^2^), of which £517 million (78%) arose from hospital admissions with procedures. £258 million (39%) of the costs attributed to excess weight were due to musculoskeletal admissions, mainly for knee replacement surgeries.

**Interpretation:**

Excess body weight is associated with increased hospital costs for middle-aged and older women in England across a broad range of conditions, especially knee replacement surgery and diabetes. These results provide reliable up-to-date estimates of the health-care costs of excess weight and emphasise the need for investment to tackle this public health issue.

**Funding:**

Cancer Research UK; Medical Research Council; National Institute for Health Research.

## Introduction

The prevalence of overweight and obesity has increased substantially in most countries in recent decades, and more than 60% of adult men and women in the UK are now overweight or obese.^1^ Excess weight is associated with an increased incidence of chronic diseases, including type 2 diabetes, vascular diseases, osteoarthritis, and some cancers, and with increased mortality.^2^, ^3^

Hospital care is the largest single component of health-care expenditure in many health systems; in England it accounts for about 44% of total National Health Service (NHS) expenditure.^4^ Studies^5^, ^6^, ^7^, ^8^, ^9^, ^10^, ^11^, ^12^, ^13^ based on individual participant data have reported strong associations between increased body-mass index (BMI) and elevated hospital costs. However, most of these studies were done on small-to-moderate numbers of participants, limiting their ability to estimate effects for different grades of obesity or the distribution of increased health-care costs across different health conditions. No reliable estimates of the effects of increased BMI on health-care costs using individual participant data are currently available for the UK. Instead, policy analyses have been informed by studies using published epidemiological data to attribute part of the prevalence and cost burden of selected conditions to obesity.^14^

We describe and quantify the relation between BMI and costs of hospital inpatient and day-case care, both overall and for specific health conditions, using individual participant data from a large cohort of women older than 50 years in England that is linked to routinely collected hospital admission data.

## Methods

### Study design and participants

Women aged 50–64 years were recruited into this prospective cohort study (the Million Women Study) in 1996–2001 through 66 UK NHS breast cancer screening clinics (60 in England). Women who were invited to breast screening were sent a study questionnaire, to be returned when they attended their screening appointment. Those who returned a study questionnaire are broadly representative of the women who attended breast cancer screening (75% of women invited).^15^, ^16^ The study questionnaire included questions about anthropometric, social, demographic, health, and other personal characteristics. BMI was derived for each participant from self-reported height and weight.

**Research in context**

**Evidence before this study**

We searched MEDLINE and Embase using terms related to economics and costs (including cost*, economic*, expenditure*, charge*), and overweight and obesity (including “obese”, “obesity”, “overweight”, “over-weight”, “over weight”, “bmi”, “body mass”), for studies published in English between Jan 1, 1990, and Sept 19, 2016, which investigated the association between overweight or obesity, or both, and direct health-care costs (appendix p 1). Several studies, mostly in the USA and Australia, investigated the relation between body-mass index (BMI) and inpatient costs in middle-aged and elderly populations, and generally reported increased costs in association with increased BMI. Two US studies in cohorts of employed individuals suggested circulatory and musculoskeletal diseases were the biggest contributors to excess costs associated with overweight and obesity. Previous analysis of the Million Women Study reported increased incidence of 19 of the 25 most common reasons for hospital admission in association with increased BMI.

**Added value of this study**

To our knowledge, this is the largest prospective cohort study, and the first in the UK, to investigate directly the associations between BMI and hospital admissions and costs, both overall and for categories of health conditions. Nearly 15% of hospital care costs among women in England aged 55–79 years were attributed to overweight and obesity, with almost all of the excess costs involving hospital procedures. Musculoskeletal conditions contributed almost 40% of the total excess weight-related costs, with knee replacement surgery the largest contributor. Diabetes is probably associated with a substantial proportion of the overall excess costs due to overweight and obesity.

**Implications of all the available evidence**

All available evidence suggests that excess bodyweight is associated with increased hospital admissions and costs, overall and for many health conditions, particularly (but not only) musculoskeletal and cardiometabolic conditions. Estimates of the associations between BMI and health-care costs are of value to health-care planners making resource allocation decisions in response to changes in population weight. The substantial additional costs attributable to excess weight in our study lend support to calls for additional investment in large-scale and cost-effective programmes to prevent and treat overweight and obesity.

Using unique NHS identification numbers and other personal details, study participants in England were linked by the Health and Social Care Information Centre (now NHS Digital) to NHS Central Registers and the Office for National Statistics (ONS) for information on deaths, cancer registrations, and emigrations, and to Hospital Episode Statistics (HES) for information on inpatient and day-case care.^17^ In HES, the information on diagnoses and procedures associated with each hospital admission were coded with WHO's International Classification of Diseases 10th revision (ICD-10)^18^ and the Office for Population Censuses and Surveys classification of surgical operations and procedures fourth revision (OPCS-4).^19^

The Oxford and Anglia Multi-Centre Research Ethics Committee provided approval for the study and all participants gave signed consent for follow-up through their medical records. Information on data access for the Million Women Study is available at the study website.

### Costs of hospital inpatient and day-case care

Each HES episode (defined as care under a particular consultant) was allocated to a health-care resource group, the classification system used to describe hospital activity in the UK. The associated costs depend primarily on any procedures done, diagnoses, hospital admission type (day-case, elective, or non-elective inpatient care), length of stay (when it exceeds a threshold specific to the health-care resource group and admission type), and patient characteristics such as age, and were calculated with 2011–12 NHS reference costs.^20^, ^21^, ^22^, ^23^ We calculated the cost of each hospital admission (in UK 2012 prices) as the sum of the costs of all episodes within the admission. Episodes were regarded as part of the same admission if they had the same admission date, had overlapping durations, or if the admission date for one episode was the same as the discharge date for another. The costs do not include outpatient attendances or costs for prescription drugs individuals were taking outside of their hospital stay.

### Categories of health conditions

Each hospital admission was allocated to an ICD-10 chapter on the basis of the recorded primary diagnosis of the admission episode.^24^ ICD-10 chapters with less than 10 000 hospital admissions during the follow-up period were combined into a category called other. ICD-10 chapter XIX (injury, poisoning, and some other consequences of external causes) was subdivided into fractures (ICD-10: S02, S12, S22, S32, S42, S52, S62, S72, S82, S92, T02), medical and surgical complications (ICD-10: T80–T88), and the remainder, which was incorporated into the existing other category. ICD-10 chapter XIII (musculoskeletal conditions) was subdivided for some analyses into arthropathies (ICD-10: M00–M25), dorsopathies (ICD-10: M40–M54), soft tissue disorders (ICD-10: M60–M79), and the remainder. Arthropathies were further subdivided into knee replacements (ICD-10: M17) and hip replacements (ICD-10: M16), identified using ICD-10 and OPCS code combinations specified by the National Joint Registry;^25^ other arthrosis (ICD-10: M15–M19 excluding knee and hip replacements with arthrosis); rheumatoid arthritis (ICD-10: M05–M06); and other arthropathies.

### Outcomes

The study outcomes were annual number of hospital admissions and annual costs (in UK 2012 prices), overall and for each category of health condition.

### Statistical analysis

Women were excluded from the analyses if they were not recruited in England, if they had a registration of cancer (other than non-melanoma skin cancer) before recruitment, were missing information on height or weight, or were underweight (BMI <18·5 kg/m^2^). Underweight women were excluded from analysis because of the substantial potential for reverse causality and residual confounding, and the small proportion of such women in the sample (<1%). Due to major changes to the hospital payment system in 2006,^20^ and to reduce the effect of confounding by pre-existing disease (ie, health conditions causing both weight change and increased health-care costs),^3^ the main analysis was restricted to hospital admissions from April 1, 2006. Women contributed person-years of data from April 1, 2006, until their date of death, emigration, or the end of follow-up for hospital admissions (March 31, 2011), whichever occurred first.

Separate estimates for annual hospital costs, annual admission rates by BMI category (18·5 to <20 kg/m^2^, 20 to <22·5 kg/m^2^, 22·5 to <25 kg/m^2^, 25 to <27·5 kg/m^2^, 27·5 to <30 kg/m^2^, 30 to <35 kg/m^2^, 35 to <40 kg/m^2^, and 40 kg/m^2^ or more),^26^ percentage increases in annual costs, and admission rates per 2 kg/m^2^ increase in BMI (a change in weight of approximately 5 kg for a woman of average height [162 cm] in England) in women with a BMI above 20 kg/m^2^ were calculated overall and for each diagnostic category with generalised linear models with a log-link function and Poisson variance. In all models further adjustments were made for age (in 5 year bands), region of recruitment (nine regions corresponding to the areas covered by the cancer registries in England), quintiles of socioeconomic status based on the Townsend deprivation index,^27^ parity (nulliparous, 1, 2, or ≥3), age at first birth (<25 years, 25–29 years, or ≥30 years), smoking (never, past, or current), alcohol intake (rarely or never, <7 units per week, or ≥7 units per week), educational qualifications (no qualifications, secondary, technical, or tertiary), HES data year, and the proportion of each HES year with contributed data (some years [<1%] were incomplete; for example, due to emigration). Missing values for any of the adjustment variables (≤5% for all variables) were assigned to a separate category for that variable. We used cluster-robust standard errors in all models to account for the lack of independence between hospital admissions of a given individual across years of follow-up. We derived variances for the estimate in each BMI category from an estimate of the variance of the log risk specific to that category, and presented them as group-specific 99% CIs.^28^ We derived standardised estimates of mean annual costs and number of hospital admissions per 1000 person-years for each BMI category using these models based on the reported characteristics of Million Women Study participants in the analysis.

In sensitivity analyses, we estimated the associations between BMI and annual hospital costs: including women with a history of cancer at baseline; using data from all HES years since 1998; excluding women with a BMI of more than 50 kg/m^2^; restricting the analysis to never-smokers because of concerns about residual confounding by smoking; excluding participants with self-reported heart disease or stroke at recruitment; and to explore the contributions of end-of-life costs, excluding the year of death and the preceding 2 years of observation for women who died during follow-up.^29^ To examine the sensitivity of estimates of percentage increases in annual costs per 2 kg/m^2^ increase in BMI to potential measurement error in BMI derived from self-reported height and weight, hospital admission rates and annual costs were additionally estimated after replacing self-reported BMI from the Million Women Study with the mean measured values of BMI within each category of self-reported BMI from the 2012 and 2013 Health Surveys for England (appendix p 2), which recorded both self-reported and measured BMI.^30^ Where estimates by self-reported BMI categories are presented in figures, they are plotted against these mean measured values, which provide a correction for both random and systematic reporting errors.

We also estimated annual costs within subgroups defined by age at the start of each annual period (<65 years, 65–69 years, or ≥70 years), smoking status (never, former, or current), alcohol intake (rarely or never, <7 units per week, or ≥7 units per week), strenuous exercise (never or rarely, or other), tertiles of socioeconomic status, and educational qualifications (no qualifications, secondary or technical, or tertiary) at recruitment. We assessed heterogeneity of proportional increases in annual costs between categories of each subgroup using a χ^2^ test.

Hospital costs attributed to overweight and obesity (BMI ≥25 kg/m^2^) were projected for all women aged 55–79 years in England, overall and for each diagnostic category. Differences in standardised mean costs between each overweight and obesity category and BMI of 20 to <25 kg/m^2^ were calculated, and then multiplied by the number of women aged 55–79 years in England in 2013 in the same BMI categories, estimated by applying the distribution of self-reported BMI among women aged 55–79 years in the combined 2012 and 2013 Health Surveys for England^30^ to the ONS mid-2013 population estimates (6·6 million women in total).^31^

The contribution of diabetes to costs attributable to excess weight is likely to be distributed across various diagnostic categories, as defined in the main analyses. Therefore, we did a secondary analysis to explore the extent to which the costs attributable to excess weight might be associated with diabetes.^6^, ^32^ The proportion of total annual costs attributed to overweight and obesity was estimated as already described but with the diabetes status added as a covariate in the annual costs model. A woman was deemed to have diabetes in an annual period if she reported diabetes at recruitment or if a diabetes diagnosis (ICD-10: E10–E14) was recorded in any hospital admission before or during that annual period. The difference in estimated annual hospital costs associated with excess weight between models with and without diabetes as a covariate was used to estimate the proportion of costs attributable to excess weight associated with diabetes.

All analyses were done with Stata 14 and R 3.2.0. Further details of methods are available in the appendix (p 12).

### Role of the funding source

The funders of the study had no role in study design, data collection, data analysis, data interpretation, or writing of the report. The corresponding author had full access to all the data in the study and had final responsibility for the decision to submit for publication.

## Results

Of the 1 198 393 women recruited into the Million Women Study in England with follow-up for inpatient and day-case care beyond March 31, 2006, we excluded 34 708 (3%) with a previous cancer registration, 59 514 (5%) with missing information on weight or height, or both, and 10 305 (1%) who were underweight (BMI <18·5 kg/m^2^). The remaining 1 093 866 women were followed up for an average of 4·9 years from April 1, 2006 (12·3 years from recruitment), during which time 1·84 million hospital admissions were recorded (table 1). Average age at recruitment was 56·1 years (SD 4·8), and 503 803 (46%) women had a BMI of <25 kg/m^2^, 394 407 (36%) were overweight (BMI 25 to ≤30 kg/m^2^), and 195 656 (18%) were obese (BMI ≥30 kg/m^2^). Overweight or obese women tended to be of lower socioeconomic status and were less likely to be current smokers, do any strenuous exercise, or drink alcohol than women with a BMI of less than 25 kg/m^2^.

**Table 1:** Baseline characteristics and details of follow-up by category of BMI

			**BMI 18·5 to <20 kg/m**^2^	**BMI 20 to <22·5 kg/m**^2^	**BMI 22·5 to <25 kg/m**^2^	**BMI 25 to <27·5 kg/m**^2^	**BMI 27·5 to <30 kg/m**^2^	**BMI 30 to <35 kg/m**^2^	**BMI 35 to <40 kg/m**^2^	**BMI ≥40 kg/m**^2^	**All women**
Number of women			30 687	173 547	299 569	239 402	155 005	139 314	41 345	14 997	1 093 866
Baseline characteristics											
	Median BMI (IQR)		19·4 (19·1–19·7)	21·5 (20·9–22·0)	23·8 (23·1–24·4)	26·1 (25·5–26·8)	28·6 (28·0–29·2)	31·7 (30·8–33·1)	36·8 (35·8–38·3)	42·7 (41·1–45·1)	25·4 (23·1–28·6)
	Mean age at recruitment (years, SD)		55·7 (4·9)	55·6 (4·8)	56·0 (4·8)	56·3 (4·9)	56·4 (4·8)	56·3 (4·8)	55·8 (4·6)	55·5 (4·6)	56·1 (4·8)
	Deprivation tertile in study population										
		Least deprived	10 677/30 440 (35%)	64 495/172 227 (37%)	108 245/297 286 (36%)	80 192/237 589 (34%)	47 669/153 840 (31%)	38 245/138 243 (28%)	9839/41 048 (24%)	3039/14 887 (20%)	362 401/1 085 560 (33%)
		Most deprived	9662/30 440 (32%)	49 029/172 227 (28%)	87 912/297 286 (30%)	77 421/237 589 (33%)	55 472/153 840 (36%)	55 278/138 243 (40%)	18 611/41 048 (45%)	7585/14 887 (51%)	360 970/1 085 560 (33%)
	Educational qualifications										
		No qualifications	10 758/29 978 (36%)	60 130/170 041 (35%)	115 886/292 888 (40%)	104 368/233 617 (45%)	73 822/150 832 (49%)	70 429/135 334 (52%)	22 070/40 116 (55%)	8465/14 561 (58%)	465 928/1 067 367 (44%)
		Secondary or technical	13 732/29 978 (46%)	81 028/170 041 (48%)	135 559/292 888 (46%)	101 182/233 617 (43%)	60 686/150 832 (40%)	51 963/135 334 (38%)	14 414/40 116 (36%)	4867/14 561 (33%)	463 431/1 067 367 (43%)
		Tertiary	5488/29 978 (18%)	28 883/170 041 (17%)	41 443/292 888 (14%)	28 067/233 617 (12%)	16 324/150 832 (11%)	12 942/135 334 (10%)	3632/40 116 (9%)	1229/14 561 (8%)	138 008/1 067 367 (13%)
	Smoking status										
		Never	14 660/29 181 (50%)	87 162/164 968 (53%)	149 313/283 739 (53%)	117 113/225 740 (52%)	74 697/145 395 (51%)	66 832/130 346 (51%)	19 678/38 575 (51%)	6868/13 892 (49%)	536 323/1 031 836 (52%)
		Former	6068/29 181 (21%)	40 544/164 968 (25%)	78 232/283 739 (28%)	65 928/225 740 (29%)	44 460/145 395 (31%)	42 192/130 346 (32%)	13 115/38 575 (34%)	5145/13 892 (37%)	295 684/1 031 836 (29%)
		Current	8453/29 181 (29%)	37 262/164 968 (23%)	56 194/283 739 (20%)	42 699/225 740 (19%)	26 238/145 395 (18%)	21 322/130 346 (16%)	5782/38 575 (15%)	1879/13 892 (14%)	199 829/1 031 836 (19%)
	Current alcohol drinkers		23 090/30 454 (76%)	139 134/172 628 (81%)	241 352/297 954 (81%)	186 281/237 929 (78%)	115 042/153 873 (75%)	96 582/138 206 (70%)	25 961/40 975 (63%)	8468/14 861 (57%)	835 910/1 086 880 (77%)
	Exercise rarely or never		4898/30 148 (16%)	23 916/171 160 (14%)	44 450/295 200 (15%)	42 903/235 618 (18%)	33 683/152 233 (22%)	36 884/136 692 (27%)	13 282/40 493 (33%)	5791/14 691 (39%)	205 807/1 076 235 (19%)
	With previous health conditions*		6526/30 681 (21%)	33 523/173 514 (19%)	62 301/299 529 (21%)	58 317/239 360 (24%)	45 148/154 984 (29%)	48 028/139 275 (34%)	17 071/41 339 (41%)	7255/14 995 (48%)	278 169/109 3677 (25%)
Details of follow-up											
	Woman-years of follow-up (1000s)		149·3	850·4	1469·8	1173·8	759·1	681·2	201·0	72·2	5356·8
	Total number of hospital admissions		47 623	248 683	454 560	397 553	281 820	277 603	93 192	37 751	1 838 785

Data are median (IQR), mean (SD), and n (%). Data shown exclude participants with missing data on characteristics. Percentage of missing data is less than 3% for all characteristics except for smoking status (5%). BMI=body-mass index.

* Any of self-reported heart disease, stroke, diabetes, rheumatoid arthritis, osteoarthritis, osteoporosis, depression, or anxiety.

**Figure 1:**
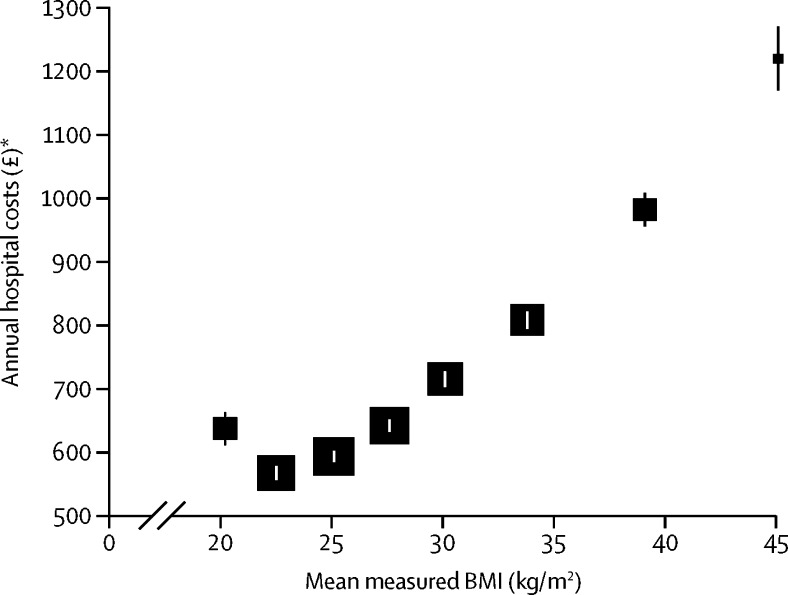
Annual hospital costs per woman by category of BMI The estimates of annual costs are adjusted for age, region of recruitment, deprivation, educational qualifications, parity, age at first birth, smoking, alcohol intake, HES data year, and proportion of HES year with contributed data. Estimates are derived by averaging predicted costs over all reported participant characteristics but with HES year set at 2010–11. Annual costs are plotted against mean measured BMI within categories of self-reported BMI from the combined 2012 and 2013 Health Surveys for England (appendix p 2). The area of each square is inversely proportional to the variance of the estimate. The error bars show 99% CI. BMI=body-mass index. HES=Hospital Episode Statistics. *In UK 2012 prices.

Rates of hospital admissions (321 per 1000 woman-years, 99% CI 316–326) and annual hospital care costs (£567, 556–577) were lowest for women with a BMI of 20 kg/m^2^ to less than 22·5 kg/m^2^ and increased steadily with increasing BMI, reaching 530 admissions per 1000 women-years (511–549) and costs of £1220 (1170–1270) for women with a BMI of 40 kg/m^2^ or higher (table 2; figure 1). For each 2 kg/m^2^ increase in BMI above 20 kg/m^2^, hospital admission rates increased by 5·0% (4·7–5·2). Annual hospital care costs were also higher (£637, 611–663) for women with a BMI of 18·5 kg/m^2^ to less than 20 kg/m^2^ than for women with a BMI of 20 kg/m^2^ to less than 22·5 kg/m^2^. For each 2 kg/m^2^ increase in BMI above 20 kg/m^2^, annual costs increased by 7·4% (7·1–7·6).

**Table 2:** Annual hospital admissions and costs by BMI

	**Hospital admissions**		**Annual hospital care costs**	
	Admissions per 1000 women-years	Difference in admission rate*	Annual costs (2012 £)	Difference in costs*
18·5 to <20	342 (331 to 354)	6·6% (3·2 to 10·1)	637 (611 to 663)	12·4% (7·9 to 17·0)
20 to <22·5 (reference)	321 (316 to 326)	0·0% (−1·5 to 1·5)	567 (556 to 577)	0·0% (−1·7 to 1·7)
22·5 to <25	334 (330 to 338)	4·0% (2·8 to 5·2)	593 (584 to 601)	4·6% (3·3 to 5·9)
25 to <27·5	356 (351 to 361)	10·8% (9·5 to 12·1)	641 (632 to 651)	13·2% (11·8 to 14·7)
27·5 to <30	383 (377 to 389)	19·3% (17·7 to 20·9)	715 (703 to 727)	26·1% (24·3 to 28·0)
30 to <35	416 (410 to 422)	29·6% (27·9 to 31·3)	808 (794 to 821)	42·5% (40·4 to 44·7)
35 to <40	473 (462 to 483)	47·1% (43·9 to 50·4)	982 (955 to 1009)	73·3% (68·9 to 77·8)
≥40	530 (511 to 549)	65·1% (59·4 to 71·1)	1220 (1170 to 1270)	115·3% (106·8 to 124·2)

Data are mean (99% CI), with floating CIs for results by BMI category. All models are adjusted for age, region of recruitment, deprivation, educational qualifications, parity, age at first birth, smoking, alcohol intake, Hospital Episode Statistics data year, and proportion of Hospital Episode Statistics year with contributed data. BMI=body-mass index.

* Differences are presented as percentage differences compared with BMI 20 kg/m^2^ to <22·5 kg/m^2^.

These results were not affected by the inclusion of women with previous cancer, the exclusion of women with a BMI of more than 50 kg/m^2^ or with previous heart disease or stroke, the exclusion of up to the last 3 years of data for women who died, or when using imputed data to account for measurement error in self-reports (appendix p 3). The inclusion of hospital admissions data before 2006 led to a marginally smaller estimated increase in costs for women with a BMI of 40 kg/m^2^ or higher. In an analysis restricted to never smokers, estimated percentage increases in costs for overweight and obesity were larger than in the main analysis. The percentage increases in annual hospital costs per 2 kg/m^2^ increase in BMI above 20 kg/m^2^ showed some statistical heterogeneity between subgroups of women; however, these differences were small in magnitude (appendix p 10).

When we extrapolated the results from the Million Women Study to all 6·6 million women aged 55–79 years (the typical age range of participants during follow-up) in England in 2013, total annual hospital costs were projected to be £4·5 billion, of which £662 million (14·6%) was attributed to overweight and obesity (BMI ≥25 kg/m^2^). Within categories of BMI, the proportion of annual costs attributed to excess weight increased from 13% (£202 million of £1529 million) among women with a BMI between 25 and 30 kg/m^2^, to 52% (£100 million of £192 million) among women with a BMI of 40 kg/m^2^ or higher (appendix p 4).

**Figure 2:**
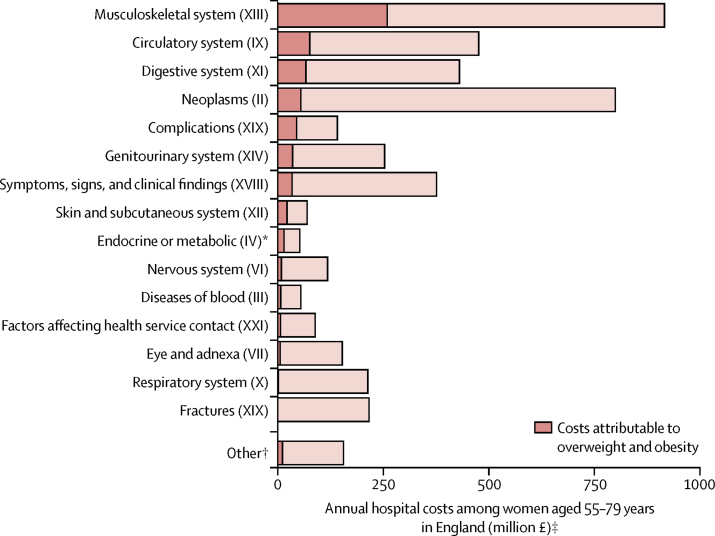
Annual hospital costs attributed to overweight and obesity among women aged 55–79 years in England by diagnostic category Overweight and obesity is defined as a BMI of 25 kg/m^2^ or more. ICD-10 chapters are ordered in the figure according to their contribution to overweight and obesity attributed costs. These estimates were derived by applying the estimates of excess costs by BMI category for each ICD-10 chapter (or combination) from the Million Women Study analysis (appendix p 11) to women aged 55–79 years in England using the Health Surveys for England 2012 and 2013 to estimate the population-level distribution of women by self-reported BMI category and UK Office for National Statistics mid-2013 population estimates (appendix p 2). We calculated excess costs relative to a BMI category of 20 kg/m^2^ to less than 25 kg/m^2^, estimated as a weighted average of the estimates of the two subcategories (20 kg/m^2^ to <22·5 kg/m^2^ and 22·5 kg/m^2^ to <25 kg/m^2^). BMI=body-mass index. *Hospital admissions were categorised by health conditions (ie, ICD-10 chapter of primary diagnosis). Although diabetes could be an underlying cause of many admissions, the categories in this figure represent the health condition for which the individual ultimately receives treatment in an inpatient setting. †All chapters with fewer than 10 000 admissions (certain infectious and parasitic diseases [I]; mental and behavioural disorders [V]; diseases of the ear and mastoid process [VIII]; pregnancy, childbirth, and the puerperium [XV]; certain conditions originating in the perinatal period [XVI]; and congenital malformations, deformations, and chromosomal abnormalities [XVII]) and the remainder of chapter XIX after the separation of fractures and medical and surgical complications. ‡In UK 2012 prices.

**Figure 3:**
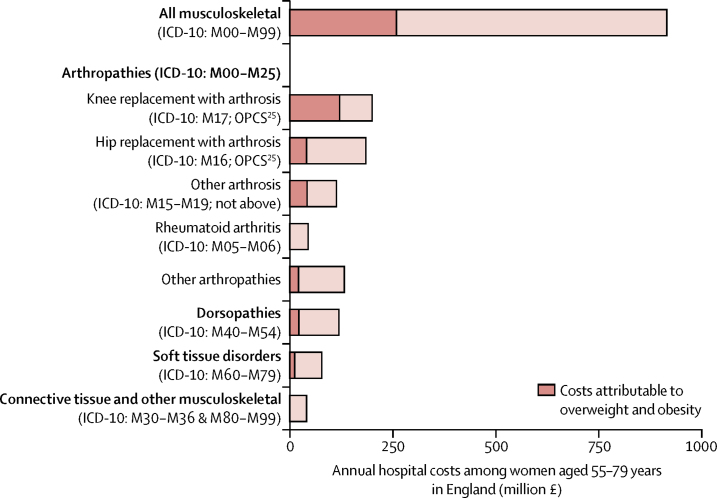
Annual hospital costs attributed to overweight and obesity among women aged 55–79 years in England by types of musculoskeletal problems Overweight and obesity is defined as a BMI of 25 kg/m^2^ or higher. These estimates were derived by applying the estimates of excess costs by BMI category for each defined subcomponent of chapter XIII (diseases of the musculoskeletal system and connective tissue) from the Million Women Study analysis to women aged 55–79 years in England using the Health Surveys for England 2012 and 2013 to estimate the population-level distribution of women by self-reported BMI category and UK Office for National Statistics mid-2013 population estimates (appendix p 2). Excess costs were calculated relative to a BMI category of 20 kg/m^2^ to less than 25 kg/m^2^, estimated as a weighted average of the estimates of the two subcategories (20 kg/m^2^ to <22·5 kg/m^2^ and 22·5 kg/m^2^ to <25 kg/m^2^). ICD-10=International Classification of Disease 10th revision. OPCS=Office for Population Censuses and Surveys classification of surgical operations and procedures 4th revision. BMI=body-mass index.

For most diagnostic categories, overweight and obesity were associated with increased hospital costs; respiratory conditions and fractures were the exceptions (figure 2; appendix p 5). Of the £662 million total annual hospital costs attributed to overweight and obesity in women aged 55–79 years in England, £517 million (78%) was attributable to admissions with procedures (appendix p 7). £258 million (39% of costs attributable to excess weight) was attributable to musculoskeletal admissions (ICD-10 chapter XIII; figure 3, appendix p 9). These costs were dominated by arthropathies (excess costs £224 million) and, particularly, knee replacement surgeries among women with osteoarthritis (£119 million). The next three ICD-10 chapters with the largest contributions to costs attributed to excess weight were diseases of the circulatory system (ICD-10 chapter IX; £80 million [12% of all costs attributed to overweight and obesity among women aged 55–79 years]), diseases of the digestive system (ICD-10 chapter XI; £70 million [11%]), and neoplasms (ICD-10 chapter II; £57 million [9%]; figure 2, appendix p 5).

27 357 (3%) of 1 093 866 women self-reported diabetes at recruitment into the study. By the end of follow-up (March 31, 2011), 69 748 (6%) women had diabetes, identified by either self-report or an ICD-10 code for diabetes recorded in any hospital admission. Using this information, the exploratory analysis suggested that diabetes might be associated with 39% of the total costs attributed to overweight and obesity.

## Discussion

Our findings show that annual hospital costs and admissions are substantially higher among overweight and obese women than among women of healthy weight (as defined by WHO).^26^ We estimated that 14·6% of all hospital care costs for women aged 55–79 years were attributed to overweight and obesity (BMI ≥25 kg/m^2^); around three-quarters of these excess costs were due to hospital admissions with procedures. Musculoskeletal conditions, especially knee replacement surgery, made the largest contribution to the costs attributed to overweight and obesity.

Our estimates of the excess annual hospital costs associated with overweight and obesity are similar to those from carefully conducted studies in populations of similar ages in other high-income countries.^5^, ^6^, ^7^ Among 225 000 middle-aged and elderly women in the 45 and Up Study^7^ in Australia, inpatient costs for obese compared with healthy weight women were 58% higher for women aged 45–64 years and 42% higher for women aged 65–79 years. Among 17 600 former industrial employees in the USA aged 65 years or older, costs were reported to be 66% higher among obese compared with healthy weight individuals.^6^ The corresponding estimate in this study was 55%. Among studies estimating costs in relation to BMI for all major types of health-care services, inpatient costs accounted for 30–50% of total overweight and obesity attributable costs.^10^, ^11^, ^12^, ^13^

The Million Women Study has previously reported that overweight and obesity is associated with higher admission rates for 19 of the 25 most common reasons for hospital admission, and longer average stays in hospital.^33^ The present study extends these findings by incorporating information on all admissions, calculating costs associated with admissions, and fully allocating costs and admissions to diagnostic categories. Overall increases in annual costs reflect both increased rates of admission and higher costs per admission (due to increased rates of comorbidities and complications, and longer admissions).^34^ In particular, we found that almost 40% of the annual hospital costs and 30% of admissions attributed to overweight and obesity were due to musculoskeletal admissions (excluding fractures). The other health conditions that contributed most to the costs associated with excess weight were diseases of the circulatory system, digestive system, and neoplasms.

Previous reports of associations between BMI and costs for types of health conditions defined by major diagnostic categories, derived from studies in the US workforce, have generally found that circulatory and musculoskeletal conditions each account for 20–30% of the additional costs associated with excess weight;^8^, ^9^ by comparison, we find that among middle-aged and older (aged 55–79 years) UK women, musculoskeletal conditions make by far the greatest contribution.

Defining categories of disease with ICD-10 chapters (derived from the ICD-10 code of the recorded primary diagnosis of the hospital admission) allows us to make tractable estimates and accords with the approach taken to programme budgeting by NHS England, thereby making the results useful to health-care commissioners. However, the full contribution of diabetes will be underestimated by the estimate for endocrine, metabolic, and nutritional disorders (ICD-10 chapter IV) because the effects of diabetes will also operate through other conditions, such as cardiovascular disease. An exploratory mediation analysis suggested that 39% of the costs attributed to overweight and obesity were associated with diabetes. However, this estimate also has limitations. Diabetes status is probably under-recorded in this study because participants who develop diabetes during follow-up are only identified if it is recorded in the HES. Conversely, any diabetes identified in a hospital record is immediately associated with the cost assigned to that record, and the likelihood that it is recorded at all can differ substantially between different conditions. The overall direction of any bias in the mediation analysis is therefore unclear.

The characteristics of Million Women Study participants were similar to those of women attending breast cancer screening during the recruitment period of the study.^15^ Women who did not attend breast cancer screening were more likely to come from more deprived areas and were less likely to have a current prescription for hormone replacement therapy, but did not differ in terms of age or recent prescriptions for various other drugs.^16^ Differences between women who participated in the study and those who did not could result in a small bias to the estimates of costs projected to all women aged 55–79 years in England, but would not substantially change our findings.

Our findings are based on observational data and despite our efforts to deal with confounding and reverse causality in main and sensitivity analyses, biases might remain, particularly for women with a very low BMI. Alternative approaches including instrumental variable methods might also be useful in addressing such issues.^35^ Additionally, BMI in the main analysis was self-reported and might underestimate true BMI.^36^ However, self-reported BMI in the Million Women Study is closely correlated with the BMI derived from measured height and weight 9 years after recruitment, and a previous study estimated that reporting errors will not result in large biases to estimates of associations over at least a decade of follow-up.^37^ Estimates in this study reflect clinical practice in England during the period of study follow-up, including clinical decisions on treatment that might be affected by weight status and comorbid conditions of obesity. In other populations or at other times, the estimated associations between BMI and costs might differ.

In conclusion, this very large prospective study was able to reliably examine the role of excess weight, not only with respect to total annual hospital costs, but also in relation to costs for categories of diagnoses. The robust findings of graded associations between categories of excess weight and increasing rates of hospital admissions and costs in England could inform health-care planning and commissioning in response to expected changes in the population weight distribution. The findings also lend strong support to calls for investment in programmes to tackle this major health problem.
