# Supplementary material for: Hospital costs in relation to body-mass index in 1·1 million women in England: a prospective cohort study
Source: Lancet Public Health. 2017 Apr 5;2(5):e214–22. doi: 10.1016/S2468-2667(17)30062-2 (PMC6196771; doi:10.1016/S2468-2667(17)30062-2)
Supplement: Supplementary appendix [file mmc1.pdf]

THE LANCET
Public Health

Supplementary appendix

This appendix formed part of the original submission and has been peer reviewed. We post it as supplied by the authors.

Supplement to: Kent S, Green J, Reeves G, et al, on behalf of the Million Women Study collaborators. Hospital costs in relation to body-mass index in 1·1 million women in England: a prospective cohort study. *Lancet Public Health* 2017; published online April 5. http://dx.doi.org/10.1016/S2468-2667(17)30062-2.

<u>Supplementary appendix to</u> "Hospital costs in relation to body mass index in 1.1 million women in England: a prospective cohort study"

Supplementary Table 1. Search terms used to identify relevant evidence before the study

Number	MEDLINE	EMBASE
1	economics/	health economics/
2	exp "costs and cost analysis" /	exp health care cost/
3	exp economics, medical/	exp fee/
4	exp economics, hospital/	budget/
5	economics, nursing/	funding/
6	economics, pharmaceutical/	cost*.ti
7	exp "fees and charges"/	economic*.ti
8	cost*.ti	(expenditure* or charge*).ti
9	economic*.ti	cost of illness/
10	(expenditure* or charge*).ti	1 or 2 or 3 or 4 or 5 or 6 or 7 or 8 or 9
11	exp "budgets"/	exp obesity/
12	cost of illness/	(obesity or obese).ti
13	1 or 2 or 3 or 4 or 5 or 6 or 7 or 8 or 9 or 10 or 11 or 12	(overweight or over-weight or over weight).ti
14	exp obesity/	(bmi or body mass index or body mass).ti
15	exp overweight/	11 or 12 or 13 or 14
16	(obesity or obese).ti	10 and 15
17	(overweight or over-weight or over weight).ti	
18	(bmi or body mass index or body mass).ti	
19	14 or 15 or 16 or 17 or 18	
20	13 and 19	

Supplementary Table 2. Distribution of women aged 55 to 79 in England in 2013 by BMI category

BMI (kg/m^2)	Percentage of women[a]	Number of women[b]	Mean measured BMI (kg/m^2)[c]
<18.5	1.7%	114,257	18.9
18.5-19.9	4.1%	273,554	20.2
20-22.4	14.2%	940,219	22.5
22.5-24.9	22.6%	1,497,925	25.1
25-27.4	20.3%	1,346,245	27.6
27.5-29.9	14.1%	930,615	30.1
30-34.9	16.0%	1,061,099	33.8
35-39.9	4.6%	302,367	39.1
40+	2.4%	157,641	45.1

BMI=body mass index

[a]The percentage of the population in each category was estimated using the 2012 and 2013 Health Survey for England based on women aged 55 to 79 with self-reported height and weight.

[b]Based on Office for National Statistics' mid-2013 population estimates there were 6,623,923 women aged 55 to 79 in England. The number of women in each BMI category was estimated by multiplying the proportions in each category from the Health Survey for England to this total population estimate

[c]Mean BMI within categories of self-reported BMI from the Health Surveys for England 2012 and 2013

Supplementary Table 3. Sensitivity analyses of the percentage increase in annual hospital costs

	Body Mass Index (kg/m^2)								
	**18.5-19.9**	**20-22.4 (reference)**	**22.5-24.9**	**25-27.4**	**27.5-29.9**	**30-34.9**	**35-39.9**	**40+**	**Trend[a]**
Base case analysis	12.4 (7.9, 17.0)	0.0 (-1.7, 1.7)	4.6 (3.3, 5.9)	13.2 (11.8, 14.7)	26.1 (24.3, 28.0)	42.5 (40.4, 44.7)	73.3 (68.9, 77.8)	115.3 (106.8, 124.2)	7.4 (7.1, 7.6)
Including pre-existing cancer	11.8 (7.5, 16.3)	0.0 (-1.7, 1.7)	4.2 (2.9, 5.5)	13.0 (11.6, 14.4)	25.6 (23.8, 27.5)	41.8 (39.8, 43.9)	72.0 (67.7, 76.4)	111.8 (103.6, 120.4)	7.3 (7.0, 7.5)
Using data from all HES years	12.0 (8.6, 15.6)	0.0 (-1.4, 1.4)	3.3 (2.3, 4.3)	11.7 (10.6, 12.8)	24.8 (23.3, 26.2)	41.7 (40.0, 43.3)	70.9 (67.5, 74.4)	101.6 (95.3, 108.2)	7.1 (6.9, 7.3)
Excluding BMI >50 kg/m^2	12.4 (7.9, 17.0)	0.0 (-1.7, 1.7)	4.6 (3.3, 5.9)	13.2 (11.8, 14.6)	26.1 (24.3, 28.0)	42.5 (40.4, 44.7)	73.3 (68.9, 77.8)	112.6 (103.9, 121.7)	7.4 (7.2, 7.7)
Never smokers only	8.4 (1.4, 15.8)	0.0 (-2.6, 2.7)	7.6 (5.6, 9.6)	18.0 (15.9, 20.2)	32.8 (29.9, 35.8)	51.6 (48.3, 54.9)	84.6 (77.8, 91.6)	119.5 (106.5, 133.2)	7.9 (7.5, 8.3)
Excluding pre-existing heart disease and stroke	12.0 (7.4, 16.8)	0.0 (-1.8, 1.8)	4.6 (3.3, 6.0)	13.0 (11.5, 14.5)	25.9 (24.0, 27.9)	41.3 (39.1, 43.6)	71.3 (66.6, 76.1)	112.6 (103.4, 122.1)	7.2 (6.9, 7.5)
Excluding year fatal events and 2 preceding years	7.7 (3.3, 12.4)	0.0 (-1.8, 1.8)	6.0 (4.7, 7.4)	16.1 (14.6, 17.6)	30.1 (28.1, 32.0)	48.1 (45.9, 50.3)	79.4 (74.7, 84.2)	117.5 (108.6, 126.8)	7.7 (7.4, 8.0)
Mean measured BMI from HSE	-	-	-	-	-	-	-	-	7.2 (6.9, 7.4)

BMI=body mass index; HSE=Health Survey for England, 2012 and 2013; HES=Hospital Episode Statistics. All models adjusted for age, region of recruitment, deprivation, educational qualifications, parity, age at first birth, smoking, alcohol intake, HES data year and proportion of HES year with contributed data.

[a]Percentage change in annual costs per 2 kg/m^2 increase in BMI (BMI $\geq$20 kg/m^2)

Supplementary Table 4. Annual hospital costs attributed to overweight and obesity among women aged 55 to 79 years in England

BMI (kg/m^2)	Number of women aged 55 to 79 in England (million)	Total annual hospital costs (£million)	Costs attributed to overweight and obesity	
			Absolute annual hospital costs (£million) (99% CI)	Proportion costs attributed (%) (99% CI)
25-29.9	2.28	1,529	202 (200-205)	13 (13-13)
30-34.9	1.06	857	239 (234-243)	28 (28-28)
35-39.9	0.30	297	121 (115-125)	41 (40-41)
≥40	0.16	192	100 (94-106)	52 (51-53)
≥25 (all overweight and obesity)	3.80	2,875	662 (643-679)	23 (23-23)

BMI=body mass index
Estimates were derived by combining estimates of annual costs per person (Table 2) and estimates of the number of women aged 55 to 79 in England by self-reported BMI category (Supplementary Table 2). See further details of methods in statistical appendix.

Supplementary Table 5. Annual costs attributed to overweight and obesity among women aged 55 to 79 years in England, by ICD-10 chapter

ICD-10 Chapter	Total annual hospital costs (£million)	Costs attributed to overweight and obesity (BMI≥25kg/m^2)	
		Annual attributed hospital costs (£million) (99% CI)	Proportion costs attributed (%) (99% CI)
Neoplasms (II)	804	57 (51-62)	7 (7-7)
Diseases of the blood (III)	56	7 (7-7)	13 (12-15)
Endocrine, metabolic, and nutritional disorders (IV)[a]	53	16 (13-18)	29 (29-30)
Nervous system (VI)	119	9 (7-11)	8 (7-8)
Eye and adnexa (VII)	156	6 (5-7)	4 (4-4)
Circulatory system (IX)	481	80 (74-85)	17 (16-17)
Respiratory system (X)	215	2 (0-3)	1 (0-2)
Digestive system (XI)	433	70 (66-74)	16 (16-17)
Skin and subcutaneous system (XII)	70	23 (20-26)	33 (32-34)
Musculoskeletal system (XIII)	914	258 (249-265)	28 (28-28)
Genitourinary system (XIV)	256	37 (33-40)	14 (14-15)
Symptoms, signs, and clinical findings (XVIII)	377	36 (33-38)	10 (9-10)
Fractures (XIX)	218	0 (0-0)	0 (0-0)
Complications (XIX)	144	47 (42-52)	33 (33-33)
Factors affecting health service contact (XXI)	88	7 (5-9)	8 (6-9)
Other[b]	159	12 (9-14)	7 (6-8)

BMI=body mass index; [a]Hospital admissions were categorised by health conditions (i.e. ICD-10 chapter of primary diagnosis). Although diabetes may be an underlying cause of many admissions, the categories here represent the health condition for which the individual ultimately receives treatment in an inpatient setting.
[b]Includes all chapters with fewer than 10,000 admissions (certain infectious and parasitic diseases [I]; mental and behavioural disorders [V]; diseases of the ear and mastoid process [VIII]; pregnancy, childbirth and the puerperium [XV]; certain conditions originating in the perinatal period [XVI]; and, congenital malformations, deformations and chromosomal abnormalities [XVII]) and the remainder of chapter XIX after the separation of fractures and

medical and surgical complications.
ICD-10=International Classification of Diseases 10th editionOverweight and obesity is defined as a BMI ≥ 25 kg/m^2. These estimates were derived by combining estimates of annual costs per person for each ICD-10 chapter (Supplementary Figure 2) and estimates of the number of women aged 55 to 79 in England by self-reported BMI category (Supplementary Table 2).

Supplementary Table 6. Annual costs attributed to overweight and obesity among women aged 55 to 79 years in England, for admissions including or not including a procedure or intervention

ICD-10 Chapter	Admissions with a procedure/intervention		Admissions without a procedure/intervention	
	**Total annual hospital costs (£million)**	**Annual attributed hospital costs (£million), (99% CI)**	**Total annual hospital costs (£million)**	**Annual attributed hospital costs (£million), (99% CI)**
All chapters (total costs)	3,501	517 (502-530)	1,042	147 (140-154)
Neoplasms (II)	727	51 (46-56)	78	5 (5-6)
Diseases of the blood (III)	36	4 (3-4)	20	4 (3-4)
Endocrine, metabolic, and nutritional disorders (IV)[a]	30	9 (7-11)	23	7 (5-8)
Nervous system (VI)	91	11 (8-14)	30	0 (0-0)
Eye and adnexa (VII)	150	6 (5-6)	5	0 (0-1)
Circulatory system (IX)	355	49 (44-53)	126	29 (26-32)
Respiratory system (X)	81	0 (0-1)	134	2 (0-3)
Digestive system (XI)	336	52 (48-55)	97	18 (16-20)
Skin and subcutaneous system (XII)	40	7 (5-8)	30	16 (13-18)
Musculoskeletal system (XIII)	865	244 (236-251)	49	13 (11-15)
Genitourinary system (XIV)	199	23 (20-25)	57	15 (12-17)
Symptoms, signs, and clinical findings (XVIII)	149	14 (11-16)	229	22 (21-24)
Fractures (XIX)	175	0 (0-0)	43	0 (0-1)
Complications (XIX)	122	40 (35-44)	22	8 (6-9)
Factors affecting health service contact (XXI)	77	5 (4-6)	11	1 (0-2)
Other[b]	71	5 (2-6)	89	7 (5-9)

[a]Hospital admissions were categorised by health conditions (i.e. ICD-10 chapter of primary diagnosis). Although diabetes may be an underlying cause of many admissions, the categories here represent the health condition for which the individual ultimately receives treatment in an inpatient setting.
[b]This includes all chapters with fewer than 10,000 admissions (certain infectious and parasitic diseases [I]; mental and behavioural disorders [V]; diseases of the ear and mastoid process [VIII]; pregnancy, childbirth and the puerperium [XV]; certain conditions originating in the perinatal period [XVI]; and,

congenital malformations, deformations and chromosomal abnormalities [XVII]) and the remainder of chapter XIX after the separation of fractures and medical and surgical complications.

ICD-10=International Classification of Diseases 10th edition. Overweight and obesity is defined as a body mass index (BMI) ≥ 25 kg/m^2. These estimates were derived by combining estimates of annual costs per person for each ICD-10 chapter, split by whether a procedure was performed in the admission event or not, and estimates of the number of women aged 55 to 79 in England by self-reported BMI category (Supplementary Table 2).

Supplementary Table 7. Annual costs attributed to overweight and obesity among women aged 55 to 79 years in England, for sub-categories of musculoskeletal disease

ICD-10 Chapter	Total annual hospital costs (£million)	Costs attributed to overweight and obesity (BMI≥25kg/m^2)	
		Annual attributed hospital costs (£million), (99% CI)	Proportion costs attributed (%), (99% CI)
All musculoskeletal (XIII: M00-M99)	914	258 (249-265)	28 (28-28)
Arthropathies (M00-M25)			
Knee replacement with arthrosis (M17; OPCS: ref #25)	200	119 (113-125)	60 (59-60)
Hip replacement with arthrosis (M16; OPCS: ref #25)	184	41 (37-43)	22 (22-22)
Other arthrosis (M15-M19; not above)	113	42 (39-45)	37 (37-37)
Rheumatoid arthritis (M05-M06)	45	0 (0-0)	0 (0-0)
Other arthropathies	135	22 (20-24)	16 (16-17)
Dorsopathies (M40-M54)	120	23 (21-26)	19 (19-20)
Soft tissue disorders (M60-M79)	78	13 (11-14)	16 (15-17)
Connnective tissue & other musculoskeletal (M30-M36 & M80-M99)	39	0 (0-1)	1 (0-2)

ICD-10=International Classification of Diseases 10th edition; BMI=body mass index.
Overweight and obesity is defined as a BMI ≥ 25 kg/m^2. These estimates were derived by combining estimates of annual costs per person for each musculoskeletal disease category and estimates of the number of women aged 55 to 79 in England by self-reported BMI category (Supplementary Table 2).

Supplementary Figure 1. Trends in annual hospital costs per 2 kg/m^2 increase in BMI above 20 kg/m^2 by category of women

	Number of women[a]	Percentage change in annual costs (99% CI) per 2 kg/m^2 change in BMI for BMI≥20 kg/m^2
**Age in years**		
<65	367,900	7.4 (7.0, 7.9)
65-70	330,565	7.7 (7.2, 8.2)
70+	364,714	6.8 (6.3, 7.3)
Heterogeneity: χ^2_3=12.0, P=0.0025		
**Smoking status**		
Never	521,663	8.2 (7.8, 8.6)
Former	289,616	7.2 (6.7, 7.7)
Current	191,376	5.6 (4.9, 6.2)
Heterogeneity: χ^2_2=79.2, P<0.0001		
**Alcohol intake**		
None	243,606	7.1 (6.6, 7.6)
<7 units per week	561,069	7.6 (7.2, 8.0)
7+ units per week	251,751	6.9 (6.1, 7.6)
Heterogeneity: χ^2_2=8.2, P=0.017		
**Strenuous exercise behaviour**		
Never/rarely	490,419	6.5 (6.1, 6.9)
Other	537,467	7.9 (7.4, 8.3)
Heterogeneity: χ^2_1=37.4, P<0.0001		
**Deprivation tertile**		
Most deprived	341,597	6.6 (6.2, 7.1)
Middle tertile	356,538	7.6 (7.1, 8.1)
Least deprived	356,985	8.2 (7.6, 8.7)
Heterogeneity: χ^2_2=35.0, P<0.0001		
**Educational qualifications**		
No qualifications	455,170	6.7 (6.4, 7.1)
Secondary or technical	449,699	8.0 (7.5, 8.5)
Tertiary	132,520	8.2 (7.3, 9.1)
Heterogeneity: χ^2_2=33.5, P<0.0001		
**All women**	1,063,179	7.4 (7.1, 7.6)

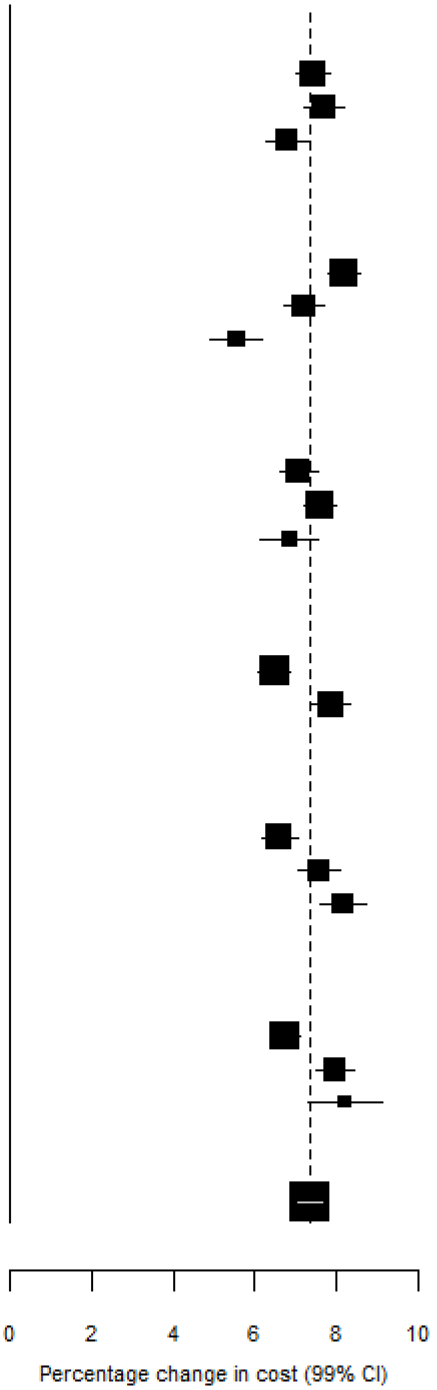

BMI= body mass index. Estimation adjusted for age, region of recruitment, deprivation, educational qualifications, parity, age at first birth, smoking, alcohol intake, HES data year, and proportion of HES year with contributed data, as appropriate. The area of each square is inversely proportional to the variance. All estimates of percentage change in annual costs have p-values<0.001. Participants with missing data on particular characteristics were excluded; the proportion of missing data is less than 3% for all characteristics except for smoking status (5%).

[a]Numbers based on women with BMI≥20 kg/m^2 only.

Supplementary Figure 2. Annual hospital costs per woman by ICD-10 chapter and category of body mass index

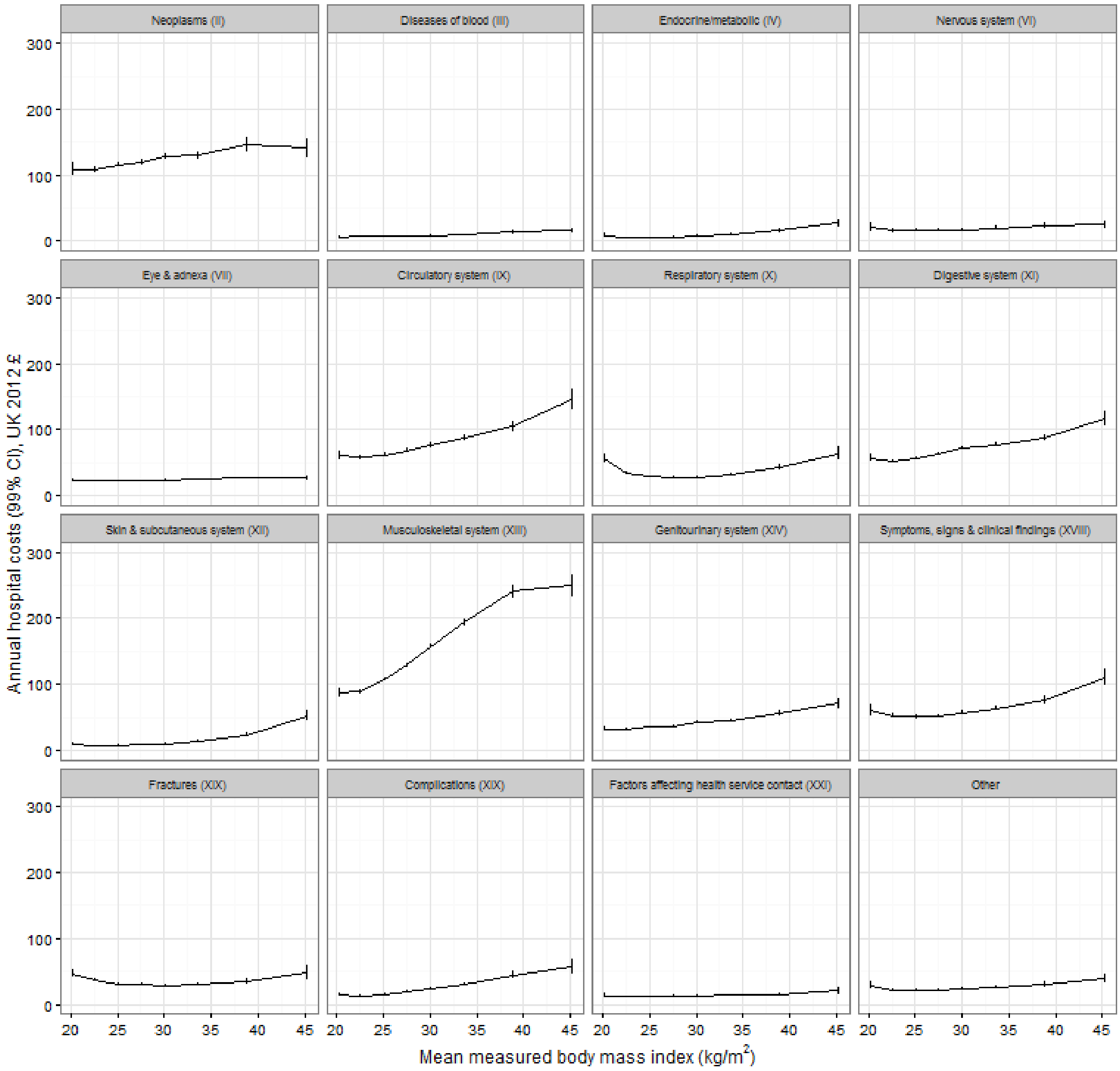

Hospital admissions were categorised by health conditions (i.e. ICD-10 chapter of primary diagnosis). Although diabetes may be an underlying cause of many admissions, the categories in Chapter IV represent the health condition for which the individual ultimately receives treatment in an inpatient setting. The 'other' includes all chapters with fewer than 10,000 admissions (certain infectious and parasitic diseases [I]; mental and behavioural disorders [V]; diseases of the ear and mastoid process [VIII]; pregnancy, childbirth and the puerperium [XV]; certain conditions originating in the perinatal period [XVI]; and, congenital malformations, deformations and chromosomal abnormalities [XVII]) and the remainder of chapter XIX after the separation of fractures and medical and surgical complications. Estimates derived from GLM log-link Poisson variance regression models with separate models were fitted for each ICD-10 chapter, adjusted for age, region of recruitment, deprivation, educational qualifications, parity, age at first birth, smoking, HES data year proportion of HES year with contributed data. Annual costs are plotted against mean measured BMI within categories of self-reported BMI from the combined 2012 and 2013 Health Surveys for England (Supplementary Table 2).

Statistical Appendix

Costing admitted patient care data

The 2011-12 NHS reference cost grouper was used to assign Healthcare Resource Groups (HRGs) to all finished consultant episodes (FCE: the unit of data in HES; refers to care under a particular consultant) across all HES years in the study. To assess the sensitivity of the study results to the version of the grouper used, we compared assignment of HRGs and corresponding costs using the 2006-07 grouper on 2006-07 HES data, but found no important differences.

Statistical modelling

Statistical model selection was performed within the generalized linear model (GLM) framework.[1] Common specification tests were used to select appropriate GLMs for the following cost outcomes: annual costs, annual costs if positive costs, and costs of admission.[2] We compared the selected models with other commonly used models in terms of metrics of fit (mean error, mean absolute error, and root mean squared error) overall and by BMI category at baseline and deciles of predicted cost (from linear regression), and in terms of predicted marginal effects for BMI. We found that single-equation Poisson regression models performed as well as two-part models for annual costs and produced very similar marginal effects. All models were estimated using cluster robust standard errors to account for the lack of independence between observations of annual costs for the same participant. Relative rates of admission and relative costs are presented in tables and figures with group-specific 99% confidence intervals, which can be compared between any pair of categories, even if neither is the reference category.[3,4]

The covariates included in the model were informed by a systematic literature review of cost studies and other identified epidemiologic studies. Demographic and physical characteristics were based on those reported by participants at recruitment into the Million Women Study. We also examined the effects of addition of each baseline variables to a minimally adjusted model consisting of BMI category, age group, region and HES year. See manuscript for final covariate specification.

Mean measured BMI within categories of self-reported BMI

The Health Survey for England (HSE) is an annual survey designed to collect information on the health and health-related behaviour of people living in private households in England. We used the 2012 and 2013 HSE data in this analysis. The 2012 and 2013 data in HSE had information on 21,313 individuals (10,333 from 2012 and 10,980 from 2013). After restricting to women aged 50-64 (the age of participants at reporting of height and weight in the MWS), the combined sample consisted of 2,087 women. Measured height and/or weight was missing for 315 (15%) of them. BMI was categorised using self-reported BMI as in analysis (see above) and means of measured BMIs within each category of self-reported BMI were derived (Supplementary Table 2).

Extrapolation of costs to women aged 55 to 79 in England

Office for National Statistics (ONS) mid-2013 population estimate for England

ONS mid-2013 estimates provide estimated population numbers by gender and age.[5] We report results for all women aged 55 to 79 years in England (N=6,623,923).

Population distribution by BMI category using data from Health Surveys for England

We separately estimated the distribution of women aged 55 to 79 in the 2012 and 2013 in categories of self-reported BMI categories among women aged 55 to 79 inclusive in the 2012 and 2013 using the appropriate year-specific survey weights provided. We then averaged across these distributions to form the distribution used for extrapolation. Supplementary Table 2 reports the estimated proportions of the population in each BMI category and absolute numbers when combined with the ONS mid-2013 population estimate.

Population projection of hospital costs and contributions of overweight and obesity

Projections are made by combining the standardised estimates of annual costs (Table 2) with the estimated population distribution by BMI. Total annual hospital costs are calculated as the sum of the products of the annual costs and number of women by BMI category. To estimate total annual hospital costs attributed to overweight and obesity we do the following. First, we calculate marginal (or attributed) costs for all BMI categories for BMI ≥25 kg/m^2 relative to a BMI of 20 to <25 kg/m^2 using the standardised estimates of annual costs. The mean cost in the healthy weight BMI group is calculated as a weighted average of costs for BMI 20 to <22.5 kg/m^2 and 22.5 to <25 kg/m^2, where the weights are the estimated numbers in the

relevant population in these BMI categories. Estimated marginal (or attributed) costs per person by BMI category (for BMI ≥25 kg/m^2) are then combined with corresponding estimates of the number of women in each category to calculate total population attributed costs by BMI category. These are added to calculate overall overweight and obesity attributed costs. Dividing this number by the total annual hospital costs provides an estimated proportion attributed to overweight and obesity.

Projected contributions of overweight and obesity to hospital costs by health condition

The process above is repeated separately for each category of health conditions (Supplementary Table 5). The estimated overweight and obesity attributed costs for each condition are divided by the total overweight and obesity attributed costs to calculate the proportion explained by each category.

To estimate the contribution of diabetes to overweight and obesity attributed costs a different approach is taken because classifying admissions according to the primary diagnosis at admission will understate the importance of diabetes, the effects of which will be distributed among other chapters. Estimate is based on previous research by the Global Burden of Metabolic Risk Factors for Chronic Disease Collaboration (BMI Mediated Effects), who estimated the extent to which the relationships between BMI, overweight and obesity and risk of coronary heart disease and stroke could be explained by metabolic mediators including glucose and diabetes status.[6] They estimated hazard ratios for the exposure-risk relationship were estimated for a model adjusted for standard confounders and for a model with additional adjustment for glucose and diabetes; the difference between the HRs in these two models gives an estimate of the proportion of the BMI-risk relationship that is explained by obesity. Here we calculate the proportion of overweight and obesity costs using standardised estimates of annual cost from a confounder adjusted model as described above. This proportion is then re-estimated from standardised estimates following additional adjustment for diabetes, where diabetes is assumed present if it is recorded in any position (using codes E10-E14) in any admission in that annual period or an earlier annual period, or was self-reported at recruitment. The proportion of overweight and obesity attributed costs explained by diabetes is then given by the difference between these two estimates.

Uncertainty in hospital cost projections

Confidence intervals for total population attributed costs by BMI category overall and by category of health conditions (presented in Supplementary Tables 5-7) were calculated as follows. First, we drew 1,000 costs by BMI category from the mean and standard errors generated from Stata's *margins* command (see above) using a normal distribution. Second, for each iteration estimates of the burden were calculated (see above for details). Confidence intervals were calculated taking the 0.5th and 99.5th percentiles as the confidence limits. These confidence intervals incorporate the uncertainty in the parameter estimates but not the uncertainty in the population which is held fixed.

Statistical Appendix References

1. Blough DK, Madden CW, Hornbrook MC. Modeling risk using generalized linear models. *J Health Econ* 1999; **18**(2): 153-71.
2. Manning WG, Mullahy J. Estimating log models: to transform or not to transform? *J Health Econ* 2001; **20**(4): 461-94.
3. Easton DF, Peto J, Babiker AG. Floating absolute risk: an alternative to relative risk in survival and case-control analysis avoiding an arbitrary reference group. *Stat Med* 1991; **10**(7): 1025-35.
4. Plummer M. Improved estimates of floating absolute risk. *Stat Med* 2004; **23**(1): 93-104.
5. Office for National Statistics. Population Estimates for UK, England and Wales, Scotland and Northern Ireland, Mid-2013. 2014. http://www.ons.gov.uk/ons/publications/re-reference-tables.html?edition=tcm%3A77-322718 (accessed 28 April 2015).
6. Global Burden of Metabolic Risk Factors for Chronic Diseases C, Lu Y, Hajifathalian K, et al. Metabolic mediators of the effects of body-mass index, overweight, and obesity on coronary heart disease and stroke: a pooled analysis of 97 prospective cohorts with 1.8 million participants. *Lancet* 2014; **383**(9921): 970-83.
